# Identification of the porcine IG-DMR and abnormal imprinting of *DLK1*-*DIO3* in cloned pigs

**DOI:** 10.3389/fcell.2022.964045

**Published:** 2022-08-10

**Authors:** Junliang Li, Dawei Yu, Jing Wang, Chongyang Li, Qingwei Wang, Jing Wang, Weihua Du, Shanjiang Zhao, Yunwei Pang, Haisheng Hao, Xueming Zhao, Huabin Zhu, Shijie Li, Huiying Zou

**Affiliations:** ^1^ Institute of Animal Sciences, Chinese Academy of Agricultural Sciences, Beijing, China; ^2^ College of Life Sciences, Hebei Agricultural University, Baoding, Hebei, China; ^3^ National Germplasm Center of Domestic Animal Resources, Institute of Animal Sciences, Chinese Academy of Agricultural Sciences, Beijing, China; ^4^ State Key Laboratory of Stem Cell and Reproductive Biology, Institute of Zoology, Chinese Academy of Sciences, Beijing, China; ^5^ Department of Human Genetics David Geffen School of Medicine University of California Los Angeles, Los Angeles, CA, United States

**Keywords:** pig, IG-DMR, DNA methylation, genomic imprinting, SCNT

## Abstract

Correct reprogramming of the *DLK1-DIO3* imprinted region is critical for the development of cloned animals. However, in pigs, the imprinting and regulation of the *DLK1*-*DIO3* region has not been systematically analyzed. The objective of this study was to investigate the imprinting status and methylation regulation of the *DLK1*-*DIO3* region in wild-type and cloned neonatal pigs. We mapped the imprinting control region, IG-DMR, by homologous alignment and validated it in sperm, oocytes, fibroblasts, and parthenogenetic embryos. Subsequently, single nucleotide polymorphism-based sequencing and bisulfite sequencing polymerase chain reaction were conducted to analyze imprinting and methylation in different types of fibroblasts, as well as wild-type and cloned neonatal pigs. The results showed that Somatic cell nuclear transfer (SCNT) resulted in hypermethylation of the IG-DMR and aberrant gene expression in the *DLK1*-*DIO3* region. Similar to wild-type pigs, imprinted expression and methylation were observed in the surviving cloned pigs, whereas in dead cloned pigs, the IG-DMR was hypermethylated and the expression of *GTL2* was nearly undetectable. Our study reveals that abnormal imprinting of the *DLK1*-*DIO3* region occurs in cloned pigs, which provides a theoretical basis for improving the cloning efficiency by gene editing to correct abnormal imprinting.

## Introduction

Somatic cell nuclear transfer (SCNT) is an assisted reproduction technology applied in the production of genetically modified (transgenic) animals, multiplication of elite animals, and protection of endangered species. Although the SCNT technology has been well developed in most domesticated and laboratory animals, the efficiency remains low, which limits its widespread application. Cloned animals show high rates of abortion during the perinatal period and reduced neonatal viability due to obesity, immunodeficiency, and respiratory defects (Ogura et al., 2013; Loi et al., 2016). A growing number of studies have demonstrated that cloned animals often undergo epigenetic modification errors, with gene imprinting being a major cause of developmental disorders in cloned animals (Matoba and Zhang 2018). The erasure and reconstruction of gene imprinting during somatic cell nuclear transfer cannot fully mimic normal gamete fertilization and embryo development, resulting in the loss of gene imprinting, which in turn affects the development of cloned embryos.

Imprinted genes are usually present in clusters, and the *delta-like homolog one* gene and the *type III iodothyronine deiodinase* gene (*DLK1-DIO3*) imprinted domains are located on chromosomes 12 (Schmidt et al., 2000) and 14 (Miyoshi et al., 2000) in mouse and human, respectively. The *DLK1*-*DIO3* imprinting domain spans a region of 825 kb in the mouse and contains multiple coding and non-coding transcripts (da Rocha et al., 2008). The main genes are the paternally expressed imprinted genes *DLK1*, *RTL1*, and *DIO3,* and the maternally expressed imprinted gene *GTL2*. The genetic spacer region DMR (intergenic DMR, IG-DMR) located between *GTL2* and *DLK1* has been demonstrated to regulate the expression of the entire DLK1-DIO3 region. The *GTL2* gene has been shown to repress the expression of the maternally expressed gene *DLK1* in cis-regulation by recruiting polycomb repressive complex II (PRC2) in mice (Zhao et al., 2010; Kaneko et al., 2014; Das et al., 2015; Sanli et al., 2018). Previous studies have reported that the control of *GTL2* expression by the IG-DMR is essential for the maintenance of imprinting in the *DLK1*-*DIO3* domain (Lin et al., 2003; Kota et al., 2014; Das et al., 2015; Luo et al., 2016).

Loss of imprinting of *DLK1*-*DIO3* is associated with severe developmental defects and malignant tumorigenesis (da Rocha et al., 2008; Jelinic and Shaw 2007; Khoury et al., 2010; Manodoro et al., 2014). The *DLK1*-*DIO3* imprinted domain plays an important role in the derivation and culture of induced pluripotent stem cells and embryonic stem cells in mice (Liu et al., 2010; Carey et al., 2011; Stadtfeld et al., 2012; Mo et al., 2015). A recent study successfully generated bimaternal and bipaternal mice by knocking out three maternal imprinting regions (including the IG-DMR) and seven paternal imprinting regions in parthenogenetic haploid stem cells and parthenogenetic haploid stem cells, respectively. The bimaternal mice survived to adulthood, while the bipaternal mice survived more than 48 h (Li et al., 2018). These results suggest that proper maintenance of imprinting in the *DLK1*-*DIO3* region is critical for embryonic development.

The expression of imprinting genes is controlled by DNA methylation marks that are established in germ cells in a sex-specific manner by cis-regulated differentially methylated regions (DMRs) called imprinting control regions (ICRs). Differentially methylated regions within imprinted loci undergo binding of specific transcription factors and modification by chromatin modifiers, ultimately establishing single allele expression patterns (Ferguson-Smith and Bourc’his 2018). Countless reports in the human and mouse have shown that the IG-DMR is the ICR of the *DLK1*-*DIO3* imprinting region. (Rocha et al., 2008; Saito et al., 2017; Tucci et al., 2019), but there are no reports in large mammals such as the pig.

Previous studies have demonstrated that the *DLK1-DIO3* region is silenced in cloned pig embryos, and the survival rate of cloned pigs is significantly improved by dosage compensation of the *RTL1* gene, indicating that the imprinting of the *DLK1*-*DIO3* region is abnormal in cloned pigs (Yu et al., 2018). Currently, there are few studies on the *DLK1*-*DIO3* region. In addition, the location of the IG-DMR imprinting regulatory region in the *DLK1*-*DIO3* region and the imprinting state of the *DLK1*-*DIO3* region in pigs remain unknown. Therefore, a detailed and systematic study of the porcine *DLK1*-*DIO3* imprinting region is necessary to provide a theoretical basis for improving the efficiency of pig cloning.

In the present study, the conserved sequences of the IG-DMRs in the mouse, human, and sheep were used for a comparative analysis of the porcine genome, and the location of the IG-DMR in the pig was localized. Methylation analysis of the IG-DMR showed that the methylation of cloned fetal fibroblasts and neonatal cloned dead pigs was higher than that of corresponding donor cells and wild-type neonates. In addition, the expression of the *DLK1*-*DIO3* region was aberrant, and the expression of *GTL2* was nearly completely lost in neonatal cloned dead pigs, indicating abnormal imprinting in the *DLK1*-*DIO3* imprinted domain.

## Materials and methods

### Primary cell isolation and culture

Porcine fetal fibroblasts (PEFs) were isolated from 20 to 30-day-old embryos of forward and backward crosses of laboratory minipigs, Duroc and Rongchang. Porcine fetal fibroblasts were cultured in Dulbecco’s modified Eagle medium containing 10% fetal bovine serum, 1% non-essential amino acids (Invitrogen), and 1% penicillin–streptomycin (Gibco) in a constant temperature and humidified incubator at 37.5 °C and 5% CO_2_.

### Collection and *in vitro* maturation of porcine oocytes

The bilateral ovaries of slaughterhouse sows were harvested within 30 min of slaughtering and dried with sterilized gauzes. The ovaries were placed in wide-mouth thermos flasks filled in sterilized saline (containing both antibiotics) at a temperature of 30–35°C and transported back to the laboratory within 2 h. The follicular fluid from follicles of 3–6 mm in diameter was collected with an 18-gauge needle connected to a filter pump in a 50-ml centrifuge tube and undisturbed for 45–60 min. The supernatant was discarded, and Poly (vinyl alcohol)-Phosphate Buffered Saline (PVA-PBS) solution was added to the precipitate, followed by resuspension in a 60-mm cell culture dish. The intact cumulus and oocytes (COCs) were collected with a mouth pipette under a body view microscope and cultured in an incubator at 38.5°C with 5% CO_2_ for approximately 40 h. The matured COCs were transferred into T2 solution containing l mg/ml hyaluronidase pre-warmed to 38.5°C, followed by gentle pipetting. The oocytes with clear perivitelline gaps and homogenized cytoplasm were selected under the body view microscope and placed in T2 solution pre-warmed to 38.5°C.

### Nuclear transfer

The mature oocytes were removed by micro-manipulation. Next, the individual donor cells were injected into the perivitelline space, and fusion was completed with two 1.2 kV/cm DC pulses (1-s interval) of 30 μs in fusion medium [0.3 M mannitol, 1.0 mM CaCl_2_, 0.1 mM MgCl_2_, 0.5 mM HEPES (pH 7.0–7.4)] using a BTX electronic cell manipulator. The oocytes were then incubated for 30 min in porcine zygote medium-3 (PZM-3), and the fusion percentage was calculated under a stereomicroscope. Fifty fused embryos were placed into a four-well dish (Nunc) containing 500 μl of PZM-3 pre-warmed to 38.5°C and incubated at 5% CO_2_ with maximum humidity.

### Embryo transfer

The day-1 NT zygotes were surgically transferred into surrogate mothers (250–300 zygotes per surrogate). Approximately 25 days later, the pregnancy status of each surrogate was determined by ultrasonography.

### Sample collection

The pregnant sows were executed on gestational day 114, and the skin, fat, muscle, and peritoneum were incised sequentially in the lower abdomen. The uterine body and uterine horns were ligated with hemostats, and the uterus was removed for dissection. An incision in the uterus was made to detach the placenta containing the fetus from the uterus. Finally, the fetus was removed for anatomical sampling. These tissues are subsequently used to extract RNA or DNA.

### RNA and DNA extraction

Fetuses and placentae were frozen in liquid N_2_ and grounded in a mortar with a pestle. Half of each tissue was used for RNA extraction, and the remaining half was used for DNA extraction. RNA was extracted using the RNeasy Mini Kit (Qiagen), according to the manufacturer’s protocol. To eliminate contamination of RNA with DNA, genomic DNA was removed by treatment with DNase I. The RNA was eluted in RNase free water and stored at −80°C. The DNA samples were extracted using the QIAamp DNA Mini Kit (Qiagen), according to the manufacturer’s protocol. The DNA was eluted in sterile RNase-free water and stored at -20 °C until use.

### RNA reverse transcription and quantitative polymerase chain reaction analysis

One microgram of total RNA was converted to a final volume of 20 μl of cDNA using the HiScript^®^III First Strand cDNA Synthesis Kit. The universal SYBR qPCR Master Mix was used for quantitative polymerase chain reactions. The procedure included 40 cycles of pre-denaturation (95°C, 30 s) and amplification (95°C, 10 s; 60°C, 30 s), followed by melting curve analysis (95°C, 15 s; 60°C, 1 min; 95°C, 15 s). The expression of the housekeeping gene glyceraldehyde-3-phosphate dehydrogenase (GAPDH) was used as a control. The primer information is provided in Supplementary Table S1. Statistical analyses were performed using Excel and graphs were prepared using GraphPad Prism 5, and the statistical significance was set at 0.05.

### Bisulfite genomic sequencing analysis

Genomic DNA was extracted from tissue samples using the TIANamp Genomic DNA Kit. For bisulfite genomic sequencing, 1 μg of gDNA was subjected to bisulfite treatment using the EpiTect Bisulfite Kit (Qiagen, Germany), according to the manufacturer’s protocol. Bisulfite sequencing PCR primers were designed using a web-based methylation primer tool (http://www.urogene.org/cgi-bin/methprimer/methprimer.cgi). The PCR products were cloned into the pMD19-T vector. At least ten randomly selected clones were sequenced. The sequences were aligned using a web-based quantification tool for methylation analysis (QUMA; http://quma.cdb.riken.jp/).

### Examination of the allelic expression status of *DLK1*, *GTL2*, and *DIO3*

All heterozygous individuals corresponding to each SNP were used to analyze the allelic expression of *DLK1*, *GTL2*, and *DIO3* by quantitative PCR. The cDNA templates were reverse transcribed from fetal and placental samples by random hexamer priming. The primers DK-F and DK-R were used for *DLK1* expression analysis, the primers GT-F and GT-R were used for *GTL2* expression analysis, and the primers DO-F and DO-R were used for *DIO3* expression analysis. The cDNA templates were amplified for 35 cycles. The PCR products were extracted from agarose gels using the Gel Recovery Kit (Sangon, Shanghai, China) and subjected to direct sequencing.

## Results

### Identification of the porcine IG-DMR

**FIGURE 1:**
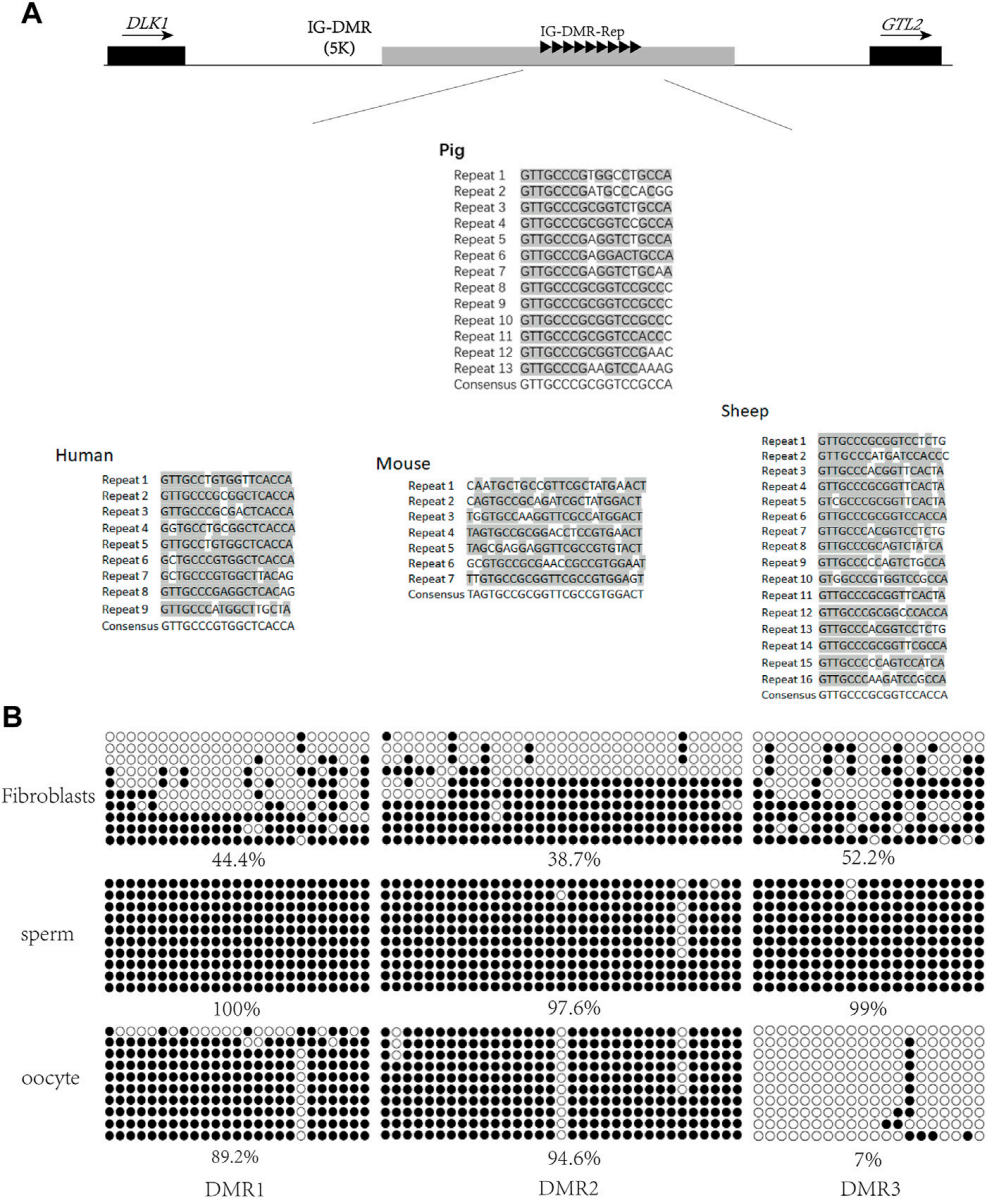
Screening of porcine IG-DMR. **(A)** Analysis of the IG-DMR tandem repeat sequences in pigs by interspecies conserved sequences. The black squares represent the positions of *DLK1* and *GTL2*, respectively. The arrow direction is the gene transcription direction. The gray rectangle represents the IG-DMR candidate region (5 kb). The black triangle represents the conserved interspecies tandem repeat sequence, and the gray areas of the tandem repeat sequences in human, mouse, sheep, and pig are the conserved sequence dinucleotides. **(B) **Analysis of methylation in porcine fibroblasts, sperm, and oocytes Each circle represents a CpG dinucleotide. The degree of methylation (%) is based on methylated CpGs/all CpGs; open circles indicate unmethylated CpGs and filled circles indicate methylated CpGs.

A previous study has reported that conserved repeats exist in the IG-DMRs of the human, mouse, and sheep (Paulsen et al., 2001). To investigate whether such repeats exist in the porcine IG-DMR, the conserved repeats from the human, mouse, and sheep were aligned with the porcine *DLK1-GTL2* region. As a result, conserved repeats were observed 5 kb upstream of *GTL2* in the pig (Figure 1A). The repeat sequence of the pig included 13 repeats, and the conserved sequence was GTT​GCC​CGC​GGT​CCG​CCA.

To map the porcine IG-DMR, we used a website tool (MethPrimer) to predict the three DMRs around the repeats and designed methylation primers to analyze this region. The methylation detection of the three DMRs was carried out in sperm, oocytes, and fibroblasts. The results showed that DMR3 was hypomethylated in oocytes, hypermethylated in sperm, and half hypermethylated and half hypomethylated in fibroblasts, suggesting DMR3 is the IG-DMR regulating imprinting in the *DLK1*-*GTL2* region (Figure 1B).

### Conserved imprinting of the porcine *DLK1*-*DIO3* region

**FIGURE 2:**
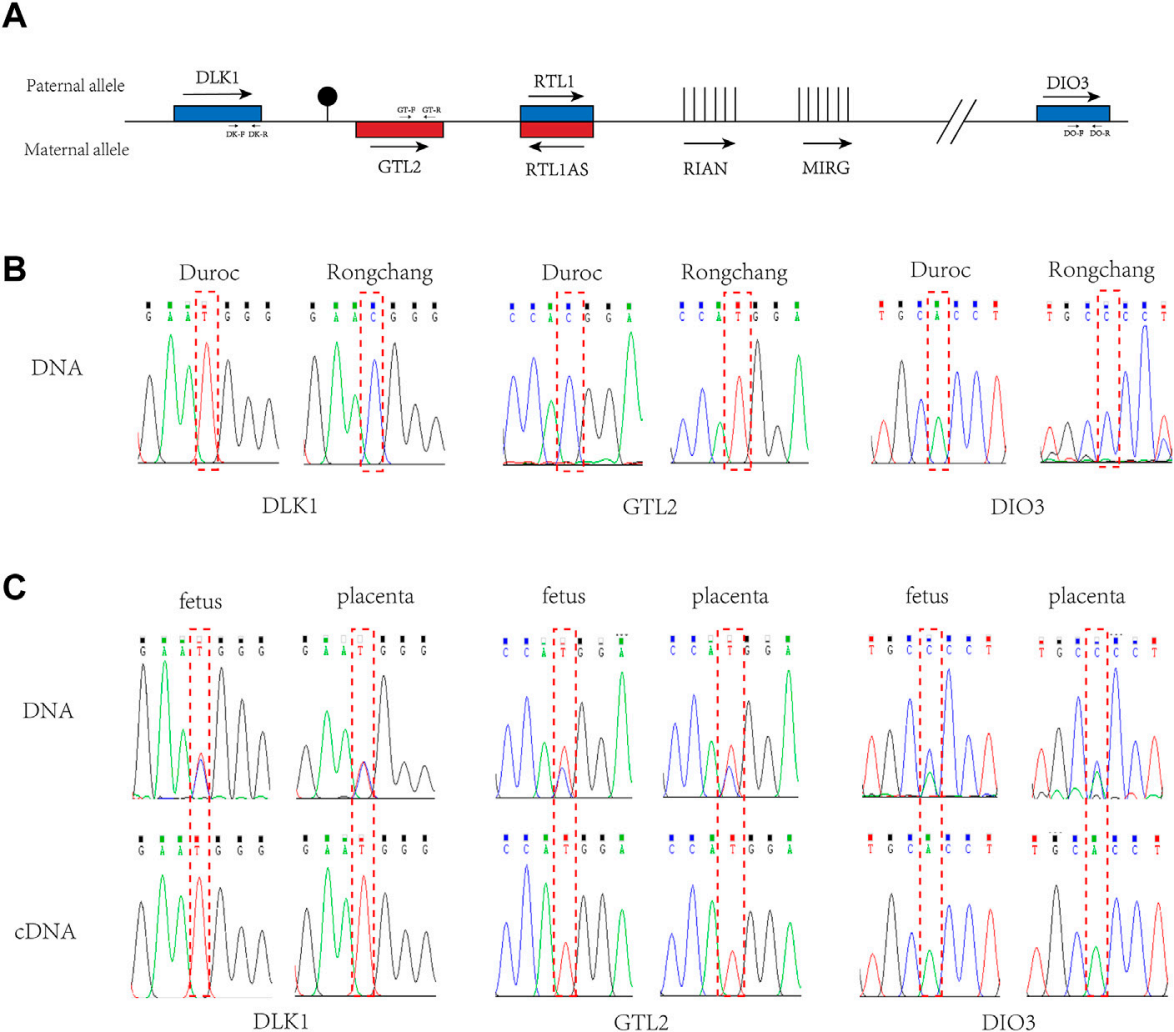
Imprinted expression of DLK1, GTL2, and DIO3 in the DLK1-DIO3 region. **(A)**. Structure diagram of the *DLK1*-*DIO3* imprinted domain. Blue squares represent paternal expression imprinted genes, and red squares represent maternal expression imprinted bases. The arrow direction is the transcription direction, and vertical lines represent non-coding RNA. Primer positions and directions are marked on the corresponding genes. **(B)**. Validation of SNPs in Rongchang and Duroc pig genomes. **(C)**. Allele-specific expression analysis of *DLK1*, *GTL2*, and *DIO3* in the fetus and placenta of the three crossbred pigs. The above sequencing results used the genome as a template for PCR, and the following sequencing results used cDNA as a template for PCR. The red-dashed boxes indicate SNP loci.

To examine the imprinting status of the *DLK1*-*DIO3* region (Figure 2A), we performed allele-specific expression analysis in 20-days (20-d) Rongchang (mother) and Duroc (father) crossbred pigs. To distinguish the expression between paternal and maternal alleles, we searched for SNPs in the *DLK1*-*DIO3* region between the two pig breeds using the UCSC database (UCSC Genome Browser Home). Three SNPs (rs81211138, rs325797437, and rs343094622) were identified in *DLK1*, *GTL2*, and *RTL1*, respectively, and verified in the genomes of the female parent Rongchang pig and the male parent Duroc pig (Table 1; Figure 2B). The SNP rs81211138 in *DLK1* was C in Rongchang and T in Duroc. The SNP rs325797437 in *GTL2* was T in Rongchang and C in Duroc. The SNP rs343094622 in *DIO3* was C in Rongchang and A in Duroc (Figure 2C). Quantitative polymerase chain reactions were performed to detect the expression of the three genes in fetuses and placentae of 20-d crossbred pigs. The PCR products were sequenced and showed monoallelic expression of *DLK1*, *GTL2*, and *DIO3* in the three crossbred pigs.

**TABLE 1:** *DLK1*, *GTL2*, and *DIO3* gene SNPs in both Rongchang and Duroc minipigs.

Gene	dbSNP	SNP position	Duroc	Rongchang
*DLK1*	rs81211138	chr7:132345395-132345395	T	C
*GTL2*	rs325797437	chr7:132161133-132161133	C	T
*DIO3*	rs343094622	chr7:130203103-130203103	A	C

Combined with SNP analysis, *DLK1* and *DIO3* were determined to be expressed by the paternal chromosome and *GTL2* was determined to be expressed by the maternal chromosome. The results indicated that *DLK1* and *DIO3* in the porcine *DLK1*-*DIO3* imprinted domain were maternally imprinted genes and *GTL2* was paternally imprinted, consistent with the imprinting status of the mouse *DLK1*-*DIO3* imprinted domain (Edwards et al., 2008; Li et al., 2008; Coster et al., 2012). These results reveal that imprinting of the *DLK1*-*DIO3* domain is conserved in mammals (Coster et al., 2012; Kalish et al., 2014).

### IG-DMR is hypomethylated in porcine parthenogenetic embryos

**FIGURE 3:**
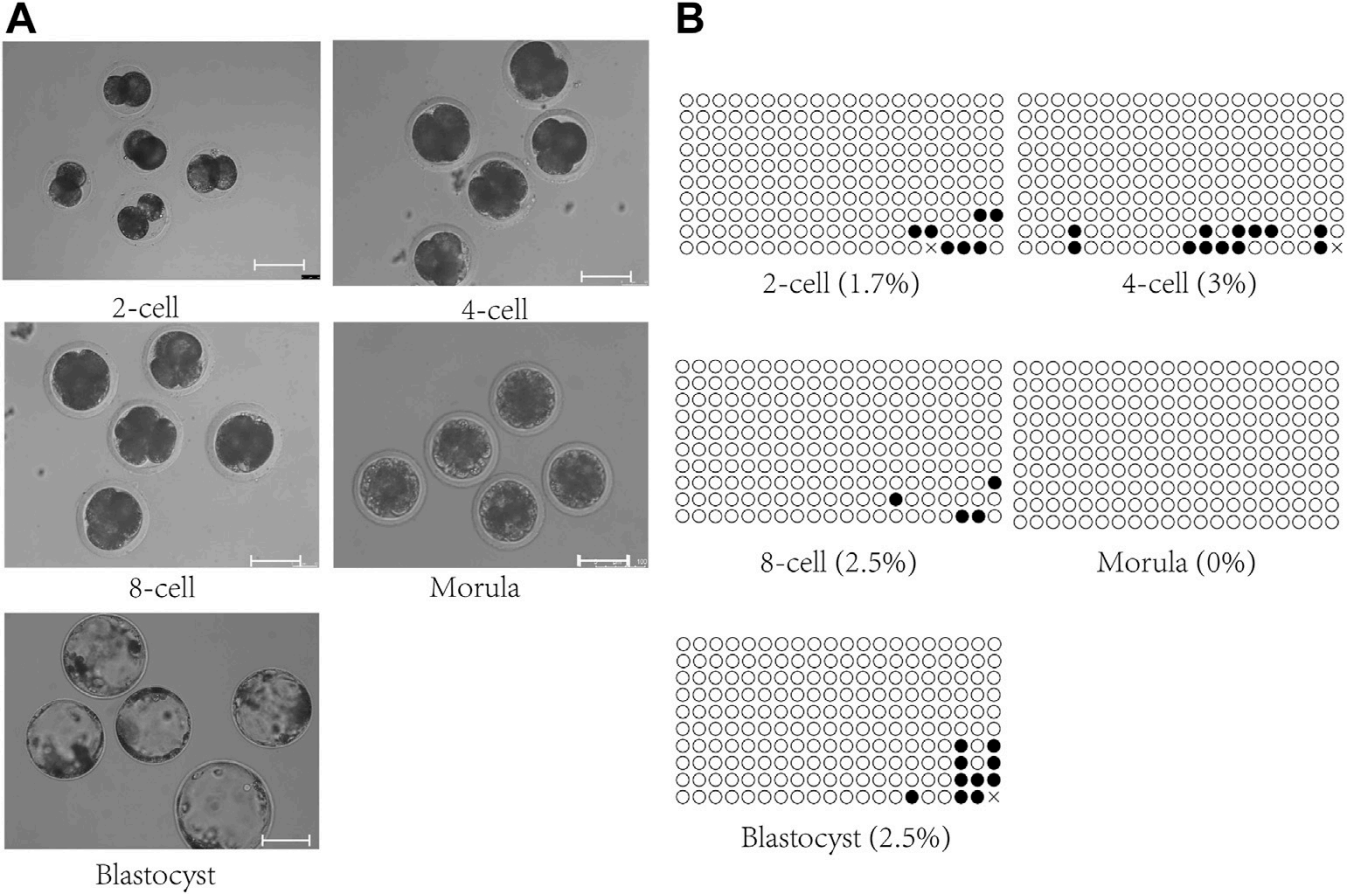
Methylation analysis of the IG-DMR in parthenogenetic embryos. **(A)**. Parthenogenetic embryos at different developmental stages. Scale bars, 100 μm. **(B)**. Methylation analysis of the IG-DMR in parthenogenetic embryos. Each circle represents a CpG dinucleotide. The degree of methylation (%) is based on methylated CPGs/all CPGs; open circles indicate unmethylated CpGs, and filled circles indicate methylated CpGs.

The genome of parthenogenetic embryos is derived from oocytes. Methylation analysis of the IG-DMR was conducted on parthenogenetic embryos at different developmental stages (Figure 3). The results showed that the IG-DMR in parthenogenetic embryos remained hypomethylated from the two-cell stage to the blastocyst stage, consistent with the hypomethylation of the IG-DMR in the female parent.

### SCNT alters imprinting and methylation of the *DLK1*-*DIO3* region

**FIGURE 4:**
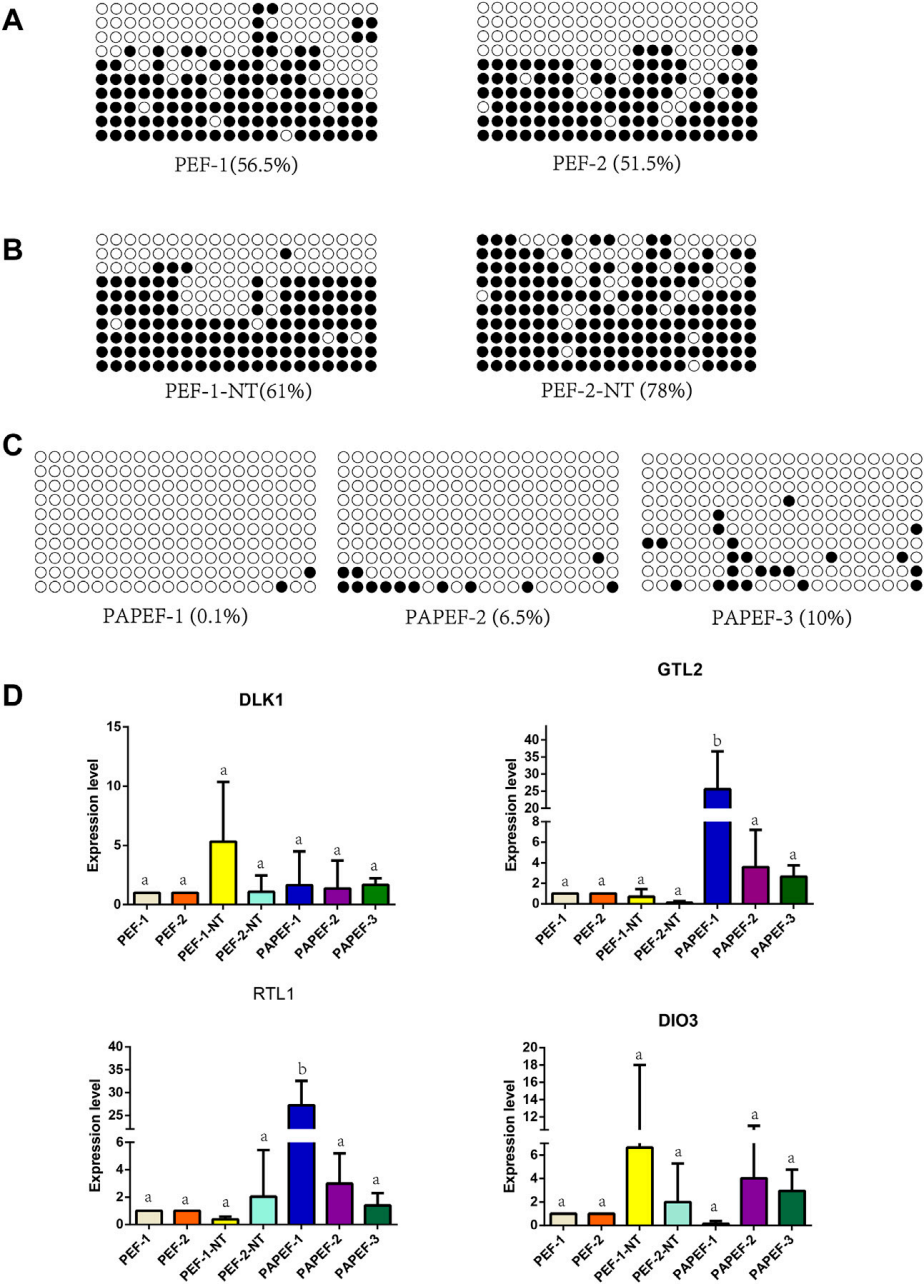
Methylation analysis of IG-DMR gene imprinting expression of the DLK1-DIO3 region in pEF, NTpEF, and PApEF. **(A)** Analysis of IG-DMR methylation levels in pEF, **(B)** NTpEF, and **(C)** PApEF. Each circle represents one CpG dinucleotide. The degree of methylation (%) is based on methylated CPGs/all CPGs; open circles indicate unmethylated CpGs, and filled circles indicate methylated CpGs. **(D)** Analysis of *DLK1*, *GTL2*, *RTL1*, and *DIO3* gene expression in pEF, NTpEF, and PApEF. Horizontal coordinates are samples, and vertical coordinates are gene expression levels. Different letters **(A,B)** indicate significant differences (*p* < 0.05).

The data from mice and some pigs showed that the methylation of the IG-DMR and the gene expression of the *DLK1*-*DIO3* imprinted domain were abnormal in unisexual embryos and cloned animals (Wei et al., 2010; Aronson et al., 2021; Wei et al., 2022). Therefore, we initially performed methylation analysis of the IG-DMR and examined gene imprinting expression of the *DLK1*-*DIO3* region in porcine fetal fibroblasts (pEF-1, pEF-2) (Figure 4A), fetal fibroblasts derived from porcine somatic cell nuclear transfer embryos (pEF-1-NT, pEF-2-NT) (Figure 4B), and fetal fibroblasts derived from porcine parthenogenetic embryos (PApEF-1, PApEF-2, PApEF-3) (Figure 4C). PEF-1 and PEF-2 were donor cells for nuclear transfer embryos of PEF-1-NT and PEF-2-NT, respectively. The IG-DMR methylation levels of PEF-1-NT and PEF-2-NT were higher than those of PEF-1 and PEF-2. The IG-DMRs of PApEF-1, PApEF-2, and PApEF-3 were hypomethylated, consistent with previous results in early parthenogenetic embryos (Sato et al., 2011). Correspondingly, the *GTL2* expression level was decreased in pEF-1-NT and pEF-2-NT, whereas it was increased in PApEF-1, PApEF-2, and PApEF-3, indicating the IG-DMR regulates the expression of *GTL2*. The expression levels of maternally imprinted genes *DLK1*, *RTL1*, and *DIO3* were not significantly different among the three types of cells (Figure 4D). These results reveal that the methylation status of the IG-DMR is abnormal in cloned and parthenogenetic embryos, which affects the expression of the paternally imprinted gene *GTL2*.

### IG-DMR is abnormally methylated in cloned pigs

**FIGURE 5:**
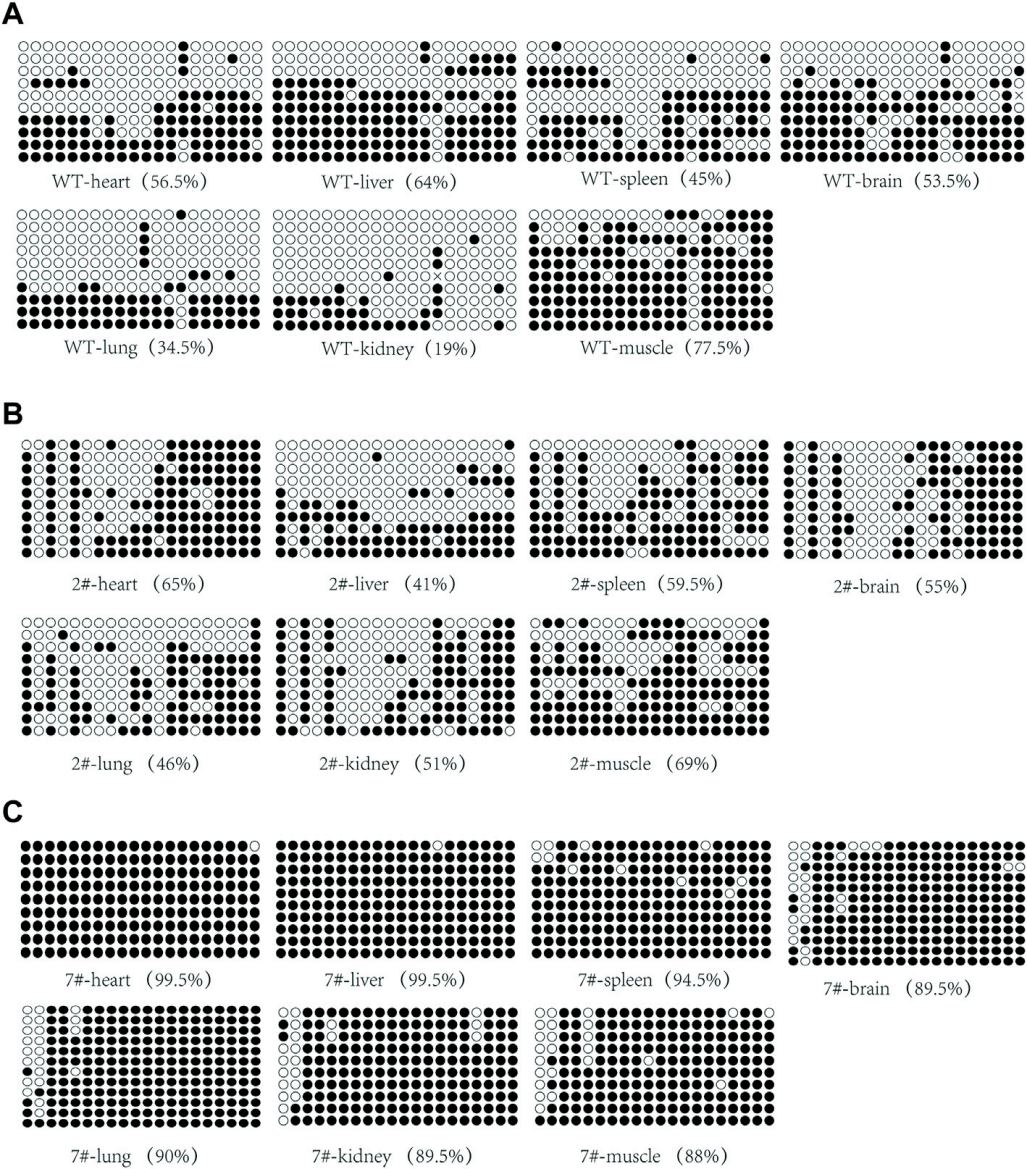
Analysis of IG-DMR methylation in wild-type neonatal pigs and cloned neonatal pigs. **(A)** Analysis of IG-DMR methylation in wild-type neonatal pigs. **(B)** Analysis of methylation in the surviving cloned piglet #2. **(C)** Analysis of methylation in dead cloned piglet #7. Each circle represents a CpG dinucleotide. The degree of methylation (%) is based on methylated CpGs/all CpGs; open circles indicate unmethylated CpGs, and filled circles indicate methylated CpGs.

To investigate in detail the methylation status of the IG-DMR in cloned pigs, we performed methylation assays on various tissues from wild-type neonatal and cloned neonatal pigs. Wild-type pigs are born naturally at term. The newborn cloned pigs were obtained by caesarean section at term, of which #7 died during birth, while #2 and #28503 survived at birth. IG-DMR methylation analysis was performed on various tissues (heart, liver, spleen, lung, kidney, muscle, and brain) from these pigs. The methylation level in cloned pig #2 was not significantly different from that in wild-type pigs (Figures 5A,B; Supplementary Figure S1A), and the methylation degree of each tissue was approximately 50%, whereas the IG-DMR in cloned pig #7 was abnormally hypermethylated, and the methylation level of each tissue was higher than 88% (Figure 5C). Therefore, we hypothesize that reprogramming errors occur in the methylation of the IG-DMR during somatic reprogramming.

### Abnormal imprinted expression in cloned pigs

**FIGURE 6:**
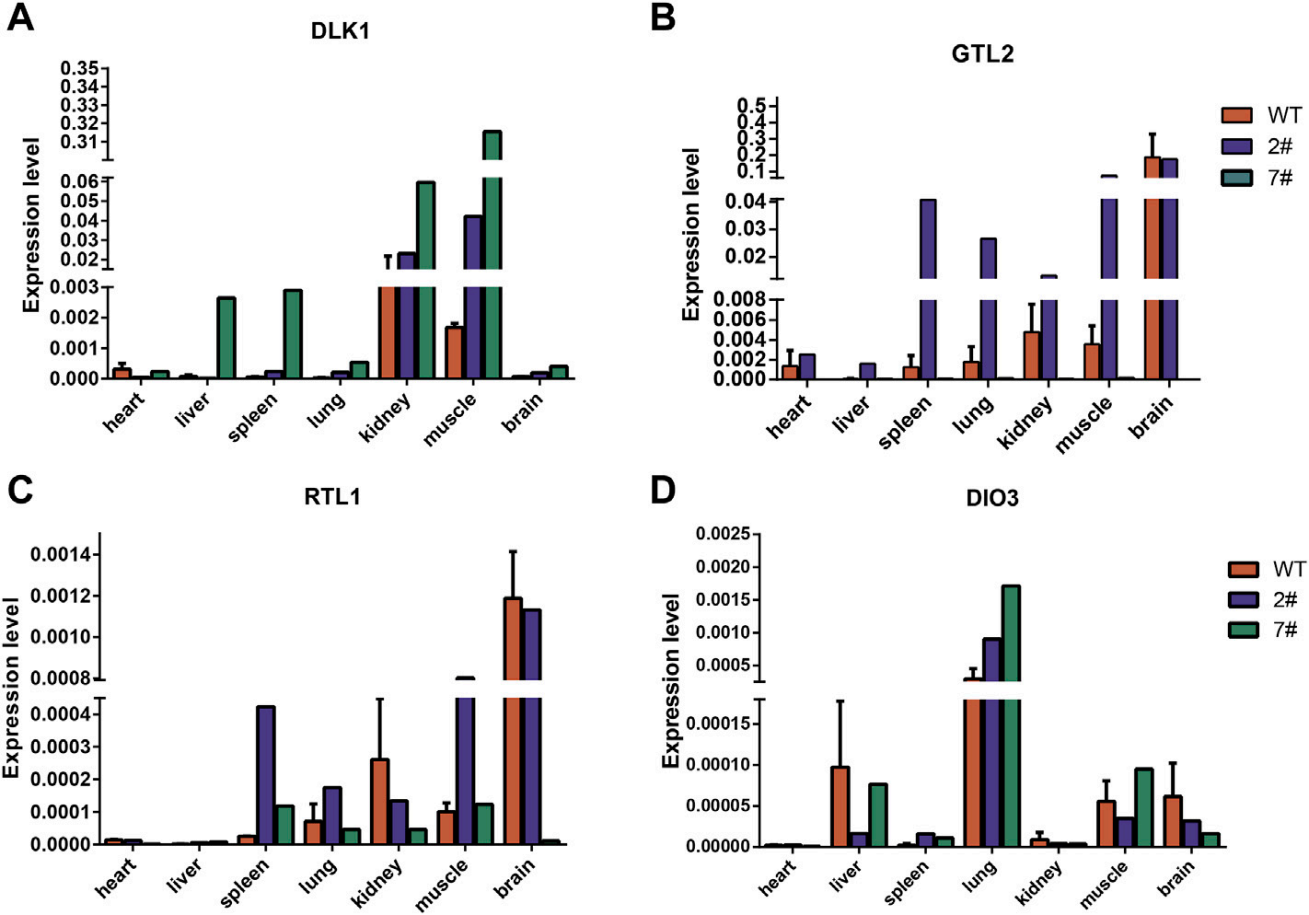
Analysis of DLK1-DIO3 imprinted domain gene expression in wild-type neonatal pigs and cloned neonatal pigs. **(A)** The expression of *DLK1* in wild-type neonatal pigs and cloned neonatal pigs. **(B)** The expression of *GTL2* in wild-type neonatal pigs and cloned neonatal pigs. **(C)** The expression of *RTL1* in wild-type neonatal pigs and cloned neonatal pigs. **(D)** The expression of *DIO3* in wild-type neonatal pigs and cloned neonatal pigs. The quantitative results are presented for tissue samples at horizontal coordinates and gene expression levels at vertical coordinates.

We analyzed the gene expression of the *DLK1*-*DIO3* imprinted domain in wild-type neonatal pigs and cloned neonatal pigs. The results of quantitative PCR showed that *DLK1* was highly expressed in the muscle (Figures 6A; Supplementary Figure S1B), *DIO3* was highly expressed in the lungs (Figure 6D), and *GTL2* and *RTL1* were highly expressed in the brain (Figures 6B,C). The expression level of *GTL2* in cloned pig #2 was higher than that in wild-type pigs, with no significant differences in the expression levels of maternally imprinted genes in most tissues. However, *GTL2* expression was nearly undetectable in cloned pig #7, and *DLK1* expression was elevated in most tissues. The hypermethylation of the IG-DMR in cloned pig #7 inhibited the expression of *GTL2*, indicating aberrant methylation of the IG-DMR was an important cause of death of cloned pig #7.

## Discussion

Genomic imprinting is critical for several life processes. The *DLK1*-*DIO3* imprinted domain is a cluster of genes essential for mammalian development. Numerous studies performed in the human and mouse have demonstrated that the IG-DMR is a key regulatory element of *DLK1*-*DIO3* and that it can also control maternally imprinted gene expression by regulating *GTL2* expression (Lin et al., 2003; Nowak et al., 2011; Kota et al., 2014; Luo et al., 2016; Wang et al., 2017; Saito et al., 2018). However, the imprinting of the *DLK1*-*DIO3* region in the pig and the location of the IG-DMR remain unknown. We examined the expression of *DLK1, GTL2*, and *DIO3* in Rongchang and Duroc crossbred pigs. The expression of male and female alleles was analyzed by SNPs in both pig breeds. The results showed that *DLK1* and *DIO3* were paternally expressed in the fetus and placenta, while *GTL2* was maternally expressed, consistent with the results observed in the human and mouse. Thus, the imprinting of the *DLK1*-*DIO3* region is well conserved in mammals.

Furthermore, we identified the tandem repeats in the pig based on the intermediate conserved repeat sequences in the human, mouse, and sheep IG-DMR and located it in the pig IG-DMR (Engemann et al., 2000; Okamura et al., 2000; Onyango et al., 2000; Paulsen et al., 2000). These tandem repeats are highly conserved, thus verifying the reliability of the pig IG-DMR locus. Nine tandem repeats were found in the human, seven conserved tandem repeats in the mouse, 16 conserved tandem repeats in the sheep, and 13 conserved tandem repeats, which spanned approximately 2.5 kb, in the pig.

The analysis of the methylation of the IG-DMR and the expression of each gene in the imprinted domain of *DLK1-DIO3* in cloned pigs showed abnormal hypermethylation of the IG-DMR in dead cloned pigs and the loss of *GTL2* expression in various tissues. *GTL2* is widely involved in various cell processes, including transcriptional repression and RNA interference-mediated mRNA degradation (Charlier et al., 2001) *GTL2* is expressed in the embryo and placenta, as well as in the adult, and the brain is the main site of its expression (Rocha et al., 2008). Maternal deletion of *GTL2* and its DMR in the mouse leads to death due to alveolar hypoplasia and hepatocyte necrosis. We speculate that the loss of *GTL2* expression in various tissues may affect the growth and development of cloned pigs.

Previous studies in the mouse have indicated that *Gtl2* suppresses the expression of the parental allele (Rocha et al., 2008). In our study, the expression levels of the maternal imprinting genes *DLK1* and *DIO3* were higher in the tissues of cloned surviving and dead pigs than those in wild-type pigs, whereas the *DLK1* expression level was significantly higher in the muscle and kidneys and that of *DIO3* was significantly higher in the lungs of wild-type pigs and cloned surviving pigs. However, the *RTL1* expression level was unchanged. *RTL1* is mainly expressed in embryonic and placental tissues, where it is essential for normal placental development, and both paternal and maternal *RTL1* deletion can lead to growth retardation and prenatal death (Yu et al., 2018). The antisense strand of the *RTL1* gene encodes an RNA transcript and two maternally expressed microRNAs that are complementary to *RTL1* (Seitz et al., 2003). We speculate that *RTL1* was not elevated like the other maternally imprinted genes in cloned dead pigs because *RTL1as*, which is expressed by the antisense strand, also participates in the regulation of *RTL1* expression.

In conclusion, we have localized the porcine IG-DMR and demonstrated that the porcine *DLK1-DIO3* region was imprinted. In addition, abnormal *DLK1-DIO3* region imprinting was observed in cloned dead pigs by methylation and quantitative PCR analyses. Our study provides a theoretical basis for the future correction of aberrant methylation in the IG-DMR by epigenetic editing and provides new directions for improving the cloning efficiency in pigs.

## Data Availability

The original contributions presented in the study are included in the article/Supplementary Material, further inquiries can be directed to the corresponding authors.
