# Supplementary material for: Identification of the porcine IG-DMR and abnormal imprinting of DLK1-DIO3 in cloned pigs
Source: Front Cell Dev Biol. 2022 Aug 10;10:964045. doi: 10.3389/fcell.2022.964045 (PMC9400927; doi:10.3389/fcell.2022.964045)
Supplement: Supplementary file 1 [file Table1.DOCX]

Table S1.List of primers used in this study.

| Primer | Sequence | Application |
| --- | --- | --- |
| DMR1-F1  DMR1-R1  DMR1-F2  DMR1-R2  DMR2-F1  DMR2-R1  DMR2-F2  DMR2-R2  DMR3-F1  DMR3-R1  DMR3-F2  DMR3-R2 | TttggagattTTtgagaaggtttga  ccaaaAatttaAAAAtAaAActacaAAtAcc  GgtTTtaagttgggattgagaaTttg  CctAAattcaaaAAAaaAAAtAcaAcc  AgtTtgTtagataTTTTaggtTtgT  CctAAAtAaaAAAtAttAaatcacaAtAaAc  GTTtgaggtTTttTagatgTTTTtg  AactcaAtAtAAcaAttttcacactA  AtaagTtgTtgggtggTttggg  AtatAatacccaActtAAcaaAcC  TgagaggaaggTagTagTaTttgTT  cctctActcaaacaAactaAaAAAc | Primers used for DMR methylation |
| DK-F  DK-R  GT-F  GT-R  DO-F  DO-R | GCTTTTCGGGCAATTTCTG  GTGAGCTCCTTCATGGATAC  taagtgtgccctctaactttt  CTCAGGACACGTAAATCAGT  ATCATCTACATTGAGGAAGCG  TTCGAGTCTCCTGCAATTC | Identification of SNPs |
| QDLK1-F  QDLK1-R  QGTL2-F  QGTL2-R  QRTL1-F  QRTL1-R  QDIO3-F  QDIO3-R | AATGGATTCTGCGAGGATGACAAT  CAAGCCCGGATGTCTAGGTC  AATGTTTGTTGATAAAGTTTGGCCG  GGAGAATAAATGAGACGGTGAGACA  Gctggagacagcgatcagag  Gaactcgtcccgtagtagcc  CGTCGACTTCCTCATCATCTACATT  TAATGGTGCCACTCTGGATGACATA | Primers used for QPCR |


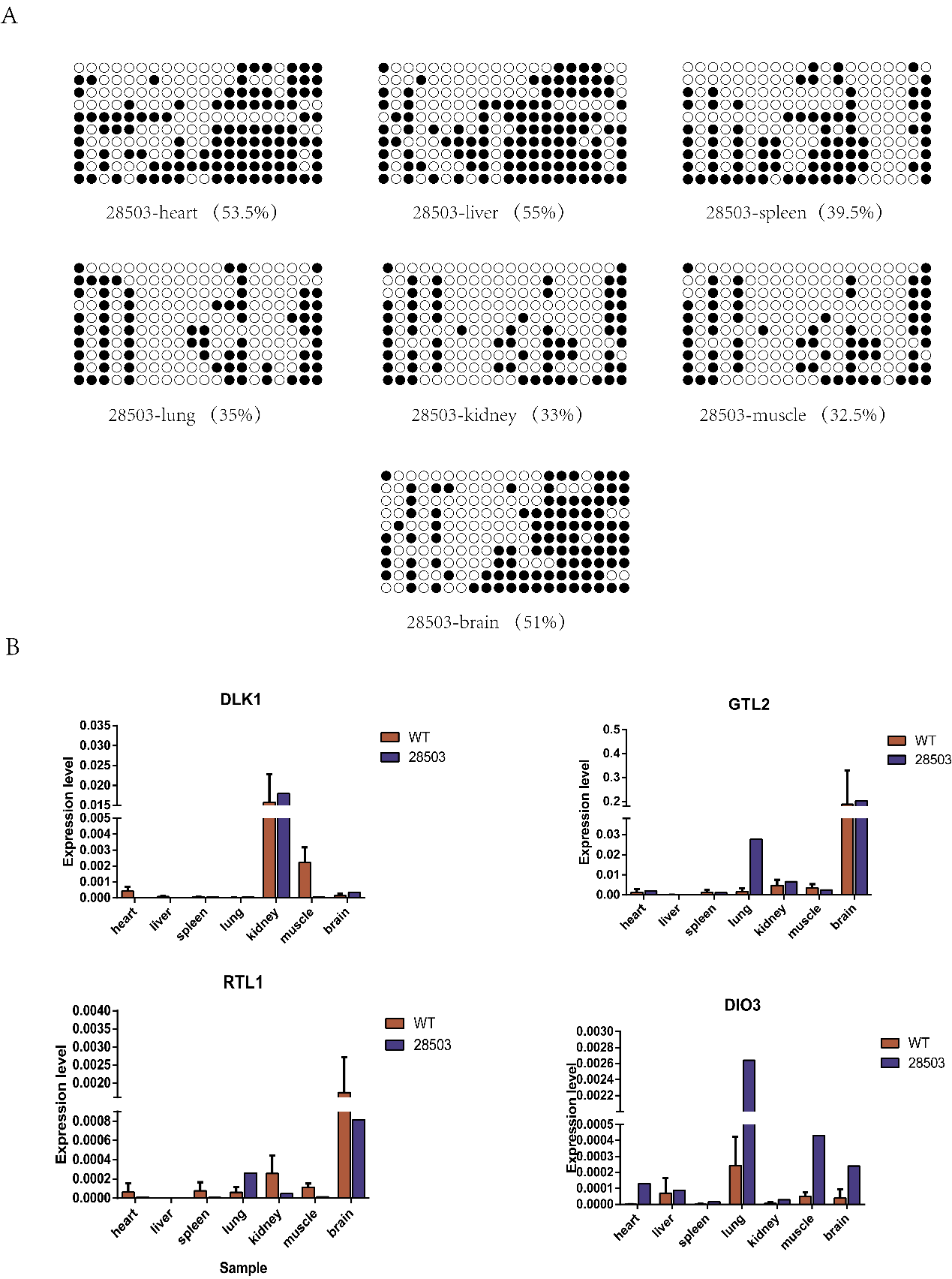


Fig S1 Analysis of methylation and gene expression by tissues of neonatal cloned pigs.

A. Analysis of methylation by tissues of the surviving cloned piglets 28503 at 114 days of gestation. B. Analysis of expression of each gene in the DLK1-DIO3 imprinted domain by tissues of the surviving cloned piglets 28503 at 114 days of gestation. Each circle represents a CpG dinucleotide. The degree of methylation (%) is based on methylated CPGS/all CPGS; open circles indicate unmethylated, filled circles indicate methylated. Quantification results are shown for tissue samples in horizontal coordinates and for gene expression levels in vertical coordinates.
